# Whole-genome sequencing illuminates the evolution and spread of multidrug-resistant tuberculosis in Southwest Nigeria

**DOI:** 10.1371/journal.pone.0184510

**Published:** 2017-09-19

**Authors:** Madikay Senghore, Jacob Otu, Adam Witney, Florian Gehre, Emma L. Doughty, Gemma L. Kay, Phillip Butcher, Kayode Salako, Aderemi Kehinde, Nneka Onyejepu, Emmanuel Idigbe, Tumani Corrah, Bouke de Jong, Mark J. Pallen, Martin Antonio

**Affiliations:** 1 Vaccines and Immunity Theme, Medical Research Council Unit The Gambia, Fajara, The Gambia; 2 Microbiology and Infection Unit, The University of Warwick, Coventry, United Kingdom; 3 Institute of Infection and Immunity, St George’s University of London, London, United Kingdom; 4 Institute of Tropical Medicine, Antwerp, Belgium; 5 Department of Medical Microbiology & Parasitology, University College Hospital, Ibadan, Nigeria; 6 National Tuberculosis Reference Laboratory, Nigeria Institute of Medical Research, Lagos, Nigeria; 7 Quadram Institute, Norwich Research Park, Norwich, Norfolk, NR4 7UA; 8 Faculty of Infectious and Tropical Diseases, London School of Hygiene & Tropical Medicine, London, United Kingdom; St Petersburg Pasteur Institute, RUSSIAN FEDERATION

## Abstract

Nigeria has an emerging problem with multidrug-resistant tuberculosis (MDR-TB). Whole-genome sequencing was used to understand the epidemiology of tuberculosis and genetics of multi-drug resistance among patients from two tertiary referral centers in Southwest Nigeria. In line with previous molecular epidemiology studies, most isolates of *Mycobacterium tuberculosis* from this dataset belonged to the Cameroon clade within the Euro-American lineage. Phylogenetic analysis showed this clade was undergoing clonal expansion in this region, and suggests that it was involved in community transmission of sensitive and multidrug-resistant tuberculosis. Five patients enrolled for retreatment were infected with pre-extensively drug resistant (pre-XDR) due to fluoroquinolone resistance in isolates from the Cameroon clade. In all five cases resistance was conferred through a mutation in the *gyrA* gene. In some patients, genomic changes occurred in bacterial isolates during the course of treatment that potentially led to decreased drug susceptibility. We conclude that inter-patient transmission of resistant isolates, principally from the Cameroon clade, contributes to the spread of MDR-TB in this setting, underscoring the urgent need to curb the spread of multi-drug resistance in this region.

## Introduction

In humans and other animals, tuberculosis (TB) is caused by a group of related mycobacterial species that form the *Mycobacterium tuberculosis* complex (MTBC) [1, 2]. The human-associated species *M*. *tuberculosis* and *M*. *africanum* form seven distinct phylogenetic lineages that are believed to have co-evolved with humans over millennia [3–7]. Lineages 2 (which includes the Beijing clade) and 4 (the Euro-American Lineage) are more widespread globally than other MTBC lineages and have been found to be more pathogenic and transmissible [8]. The Cameroon clade from Lineage 4 is the most common genotype of *M*. *tuberculosis* isolated from patients with active pulmonary tuberculosis in parts of West Africa including Nigeria [9–13]. A unique feature of MTBC in West Africa is the presence of two *M*. *africanum* lineages, human-associated ecotypes that cause up to 40% of tuberculosis in parts of West Africa [14].

Although mortality attributed to TB has halved since 1990, it remains a leading cause of death by infectious disease globally [15]. In 2015, there were an estimated 10.4 million new cases of, TB globally, resulting in 1.5 million deaths [16]. Nigeria is in the top-20 list for TB, TB/HIV and MDR-TB burden [17–19]. It was estimated that in 2012 only 3% cases of MDR-TB that occurred in Nigeria were detected and reported [20]. Diagnosis of MDR-TB is challenging because traditional phenotypic approaches to sensitivity testing in tuberculosis are slow and laborious. Bacterial whole-genome sequencing (WGS) offers early detection of resistance to both first- and second-line drugs, making it useful for diagnosing and guiding treatment of MDR-TB [21, 22].

In high-burden settings such as Nigeria, inter-patient transmission is the main source of MDR-TB infection [23]. Studies have employed WGS to infer transmission of *M*. *tuberculosis* by combining genetic distances (as a measure of single nucleotide variants), epidemiological data and/or phylogenetic data [24]. The SNV threshold for inferring transmission varies with location and the diversity of the dataset [24]. In 2013, Walker and colleagues proposed that isolates from epidemiologically linked patients that differed by 5 single nucleotide variants (SNVs) or fewer were indicative of inter-patient transmission of TB [25]. Although some studies have limited inference of transmission to isolates that differ by as few as 1,2 or 3 SNVs [26–28], Guerra-Assunção and colleagues inferred transmission from epidemiologically linked cases between isolates differing by as many as 10 SNVs [29].

Here, we present whole-genome analyses of MTBC isolates that were collected from two South-West Nigerian TB referral centers as part of the WANETAM MDR-TB surveillance. Our results shed light on the genomic changes underlying drug resistance and place these isolates within a phylogenetic context in the MTBC. Our study represents a proof-of-concept to demonstrate the feasibility of using WGS to gains insights into the transmission and evolution of MDR-TB in West Africa.

## Methods

### Isolates and antimicrobial susceptibility testing

Between December 2011 and July 2014, 177 MTBC isolates were sent to the MRC Unit the Gambia (MRCG) from tertiary tuberculosis referral centers at two sites in South-West Nigeria: The Nigerian Institute of Medical Research, Lagos and the University College Hospital, Ibadan. These isolates were recovered from patients with active pulmonary TB that reported to the referral centers between 2011 and 2014. Isolates were tested for viability and anti-microbial susceptibility at MRCG [30]. Break-point susceptibility testing for the first-line drugs streptomycin (STR, 1 μg/mL), isoniazid (INH, 0.1 μg/mL), rifampicin (RIF, 1μg/mL), and ethambutol (EMB, 5 μg/mL) was done using the MGIT 960 system (Becton Dickinson, Sparks, MD, USA) according to manufacturer’s instructions [31]. MDR strains were further tested using the MGIT system for susceptibility to kanamycin (KAN, 2.5 μg/mL) capreomycin (CAP, 2.5 μg/mL), ofloxacin (OFX, 2 μg/mL), and ethionamide (ETH, 5 μg/mL) (Sigma-Aldrich, St. Louis, Mo, USA) according to WHO guidelines [32].

WGS was performed on 75 MTBC isolates (43 MDR and 32 non-MDR) from 177 stored isolates. TB patients recruited at the Nigerian Institute of Medical Research were sampled at multiple time points during treatment. Therefore, for some patients, two or three isolates obtained at different time points were sequenced. The dates presented show when the samples were received at the MRCG. Thus, in some cases it was possible to infer the order of sampling based on sample reception date, even though the exact date of sample collection could not be established. The Joint Gambia Government/MRC Ethics Committee, the University of Ibadan and University College Hospital, Ibadan Joint ethical Review Committee and the Nigerian Institute for Medical Research Institutional board approved the study protocols.

### Genomic DNA extraction and sequencing

Genomic DNA was extracted from Middlebrook 7H9 liquid cultures that had been incubated for 5–8 weeks using acetyltrimethyl ammonium bromide (CTAB) method [33]. Genomes were sequenced on the MiSeq with the V2 reagent kit (2 x 250 bp) following Nextera XT library preparation according to the manufacturer’s instructions (Illumina, Little Chesterford, UK). Sufficient coverage (>10X) could not be obtained on two isolates, so the final dataset contained 73 isolates of MTBC.

### Phylogenetic analysis, drug susceptibility and lineage determinants

The sequencing reads were mapped to the *M*. *tuberculosis* H37Rv reference genome and variant sites were called as previously described [34]. Briefly, variants were called on sites that were covered by at least two reads in each direction, had a Q30 mapping score > 30, and were supported by > 75% of the reads. The maximum likelihood phylogeny was reconstructed from SNVs in the core genome with RAxML using a general time-reversible model of substitution [35]. The phylogenetic tree was visualized with FigTree and manually annotated.

The user-friendly web tools Kvarq, Phyrese and TBProfiler were used to infer phylogenetic lineage from sequencing reads [36–38]. Kvarq classified samples into the seven global lineages, while Phyrese and TBprofiler offered more highly resolved classification of strains into the sub-lineages of the Euro-American super-lineage, Lineage 4 (S1 Table). All three tools reported known resistance mutations (S2 Table).

## Results

Genome sequences from 73 clinical isolates of MTBC, obtained from 63 TB patients were analysed (Table 1). Isolates from the same patient taken at different times were classed as a single clinical case. Isolates were mapped to the H37Rv reference genome with an average coverage of 49.6x (range 24.3 to 78.4). The phylogeny was reconstructed from 8667 variant core genome sites, excluding known repetitive regions and regions of deletion.

**Table 1:** Summary of patient metadata for 63 patients included in the study.

		Ibadan		Lagos	
		MDR	Non-MDR	MDR	Non-MDR
**Total**		6	15	30	12
**HIV Status**	Negative	6	15	21	8
	Not Done			2	
	Positive			7	4
**Gender**	Female	4	3	11	6
	Male	2	12	18	6
**Treatment History**	Failure			2	3
	New		8	4	4
	Relapse			2	4
	Retreatment	6	7	21	1
**Age (years)**	0–15		1	1	1
	16–30	1	6	10	1
	Above 30	5	8	18	10

### Phylogenetic analysis

**Fig 1:**
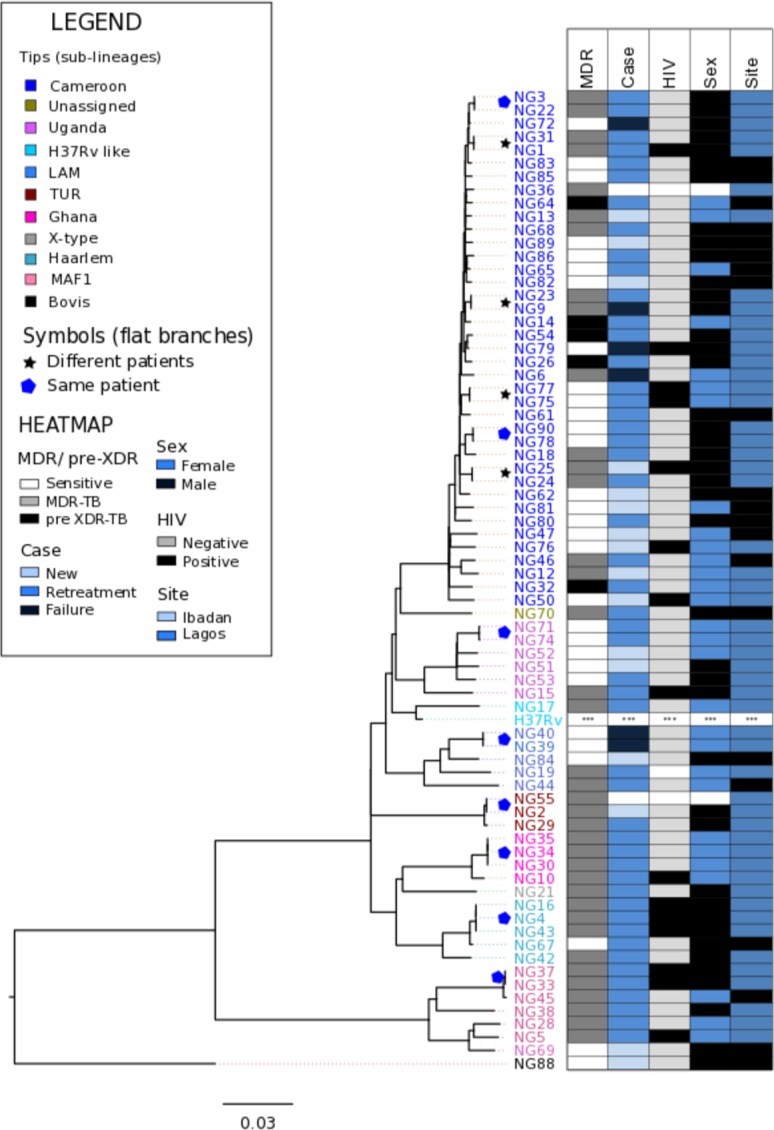
Maximum likelihood phylogeny of *Mtb* isolated from patients with active pulmonary TB. Branches are coloured by lineage and tips are coloured by the sub-clades within the lineages. A star marks almost identical genomes (<3 SNVs difference) isolated from different patients. Adjacent data links strains to patient metadata: MDR and pre-XDR status, treatment status, HIV status, sex and recruitment site and date of sample reception.

The Euro-American super-lineage (lineage 4) was predominant, found in 56 cases (88.9%). The Cameroon sub-lineage of lineage 4 was associated with 37 cases (59%: 52% and 67% of MDR and non-MDR cases respectively) (Table 2). Our tree topology (Fig 1) places our isolates from the Cameroon sub-lineage in a monophyletic clade, with an average within-clade core-genome pairwise SNV distance of 97 (range: 1–195). Other genotypes from lineage 4, including the H37Rv, LAM, TUR, Ghana, Haarlem and X-type sub-lineages, accounted for 20 cases (32% of cases).

**Table 2:** Summary of the prevalence of MDR within the different clades among 63 patients.

Lineage	Clade	MDR	Non-MDR
*M*. *bovis*	Bovis		1
Lineage 4	Cameroon	19	18
	Uganda	2	4
	Ghana	2	
	X-type	1	
	H37Rv	1	
	Haarlem	2	1
	LAM	2	2
	TUR	2	
Lineage 5	West Africa 1	5	1
	**Grand Total**	**36**	**27**

Six patients (9.5% of cases) were infected with isolates from the *M*. *africanum* sub-lineage 1 (MAF1), also called the West Africa 1 lineage or Lineage 5. Two serial MAF1 isolates recovered from a retreatment cases from Lagos were identical. However, these isolates differed from an isolate recovered from a retreatment case in Ibadan by 16 SNVs in the core genome. Although this is above the threshold for recent inter-patient transmission, this observation suggests that the MAF1 lineage has been involved in transmission of MDR-TB in the recent past. One patient was infected by an isolate belonging to *M*. *bovis*, a lineage usually associated with animal infection, which was placed as an outlier on the phylogenetic tree.

### Microevolution: Genomic changes during treatment and transmission

**Fig 2:**
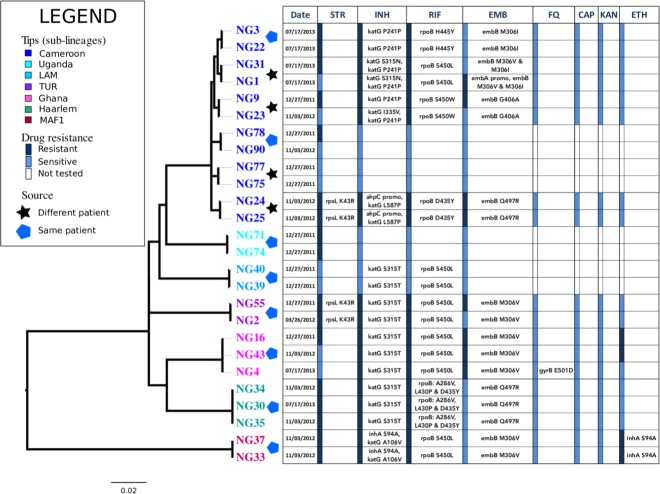
Microevolution of *Mtb* within patients during the course of treatment. Phylogenetic tree is linked to resistance profile of isolates that were paired with at least one near-identical genome (<3 SNVs difference). These genomes represent isolates from patients that were samples at least twice during the course of treatment as well as almost identical genomes from different patients. The resistance profiles for Streptomycin (Str), Isoniazid (INH), Rifampicin (RIF), Ethambutol (EMB), Flouroquinolones (FQ), Capreomycin (CAP), KANAMYCIN (KAN) and Ethionamide (ETH) are shown. The presence of resistance mutations to each drug are listed next to a bar indicating phenotypic susceptibility.

We report two scenarios where closely related pairs of isolates were recovered from different patients and one of the isolates had acquired resistance mutations (Fig 2). The MDR strains NG9 and NG23 were isolated from two patients that were enrolled in Lagos in 2011 and 2012, respectively. These strains differed at a single nucleotide site in their core genomes: the NG23 isolate has acquired the *katG* mutation L587P. Similarly NG1 and NG31 were near-identical MDR strains isolated from different patients. Both isolates harbored canonical mutations in the *embB* codon 306. However, NG1 acquired a further mutation in the *embA* promoter region and consequently NG1 was ethambutol-resistant, while NG31 was susceptible to ethambutol according to phenotypic testing.

An MDR strain belonging to the Ghana genotype was isolated from a patient who was enrolled in 2011 for retreatment. Strains NG16, NG43 and NG4 were isolated from this patient on successive hospital visits. All three strains were susceptible to fluoroquinolones on phenotypic testing, but the final isolate sampled from the patient in 2013 (NG4) had acquired a mutation associated with low-level fluoroquinolone resistance (*gyrB* E501D) and should therefore be classified as pre-XDR TB according to its genotype (Fig 2).

### Molecular mechanisms propagating multidrug and pre-extensively drug resistant TB

**Fig 3:**
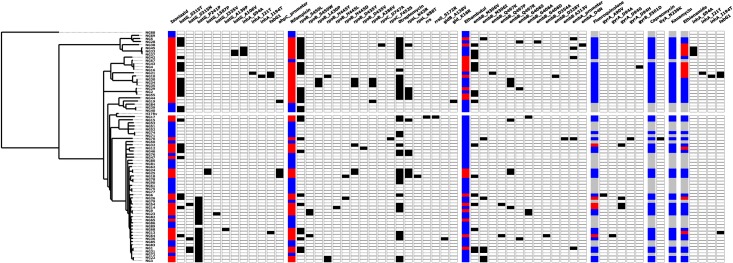
Molecular mechanisms of resistance to first- and second-line anti-TB drugs. A heatmap showing the phenotypic susceptibility profiles to first- and second-line anti-TB drugs and the presence of known resistance mutations. Blue = phenotypically susceptible, Red = phenotypically resistant, Black = presence of resistance mutation, white = absence of resistance mutation and grey = no phenotypic testing.

MDR was present in 39 clinical cases, while three clinical cases presented with resistance to isoniazid but not to rifampicin. Looking for known resistance mutations in the genome sequence provided insights into the molecular mechanisms causing multidrug resistance in our dataset (Fig 3). At least one known isoniazid-resistance mutation was found in 35 out of 39 clinical cases where isoniazid resistance was reported from phenotypic susceptibility testing. Thirty-three cases of isoniazid resistance were associated ≥1 resistance mutations in *katG or inhA*. Mutations at codon 315 of *katG* were present in 21 isoniazid resistant clinical cases and one case reported as isoniazid-sensitive. Mutations in the *inhA* and *ahpC* promoter regions were combined with other resistance mutations in some isoniazid resistant strains, while a mutation in *ahpC* was the only resistance mutation found in two MDR clinical cases.

All isolates from rifampicin-resistant clinical cases carried at least one resistance mutation in the *rpoB* gene. In two clinical cases, a mutation in the *rpoB* gene was complemented by an apparent epistatic mutation in the *rpoC* gene. At least one mutation in the *embCAB* operon was observed in 30 out of 34 clinical cases of MDR. At least one resistance mutation in the *embCAB* operon was found in 13 out of 16 (81%) ethambutol-resistance cases, while 17 out of 44 (39%) sensitive cases were associated with at least one mutation on the operon. Mutations at codons 306, 497 and 406 of the *embB* gene were detected among both ethambutol-resistant and ethambutol-sensitive isolates. Only 12 out of 29 (41.4%) cases of streptomycin resistance were associated with a known resistance mutation.

Five clinical cases of pre-extensively drug resistant (pre-XDR) were associated with fluoroquinolone resistance based on phenotypic testing. All five clinical cases of pre-XDR-TB occurred among retreatment cases, were associated with isolates from the Cameroon clade that were linked to a mutation in the *gyrA* gene. Notably, *gyrA* mutations that confer low-level fluoroquinolone resistance were present in two fluoroquinolone-susceptible MDR isolates (NG6 and NG70),. One of these isolates (NG70) also harbored the *tlyA* (Rv1694) N236K mutation that confers resistance to capreomycin. This isolate would be classified as extensively drug resistant tuberculosis (XDR-TB) on grounds of genotype, but was apparently sensitive to all second line drugs by phenotypic testing. Of nine ethionamide-resistant isolates, five were linked to at least one mutation in the *inhA* gene or the *inhA* promoter region FABG1.

## Discussion

The high prevalence of Cameroon clade strains in this dataset (59%) is similar to recent estimates from Nigeria based on spoligotyping [12]. There is evidence that the Cameroon lineage has been spreading within the community. Within the Cameroon clade, there were four instances where closely related genomes (< 3 SNVs difference) were recovered from two different patients (marked by * on Fig 1). The SNV difference between these pairs of isolates falls within the threshold for inter-patient transmission set by Walker and colleagues [25].

There was less evidence of ongoing transmission among other Euro-American sub-lineages, since isolates from different patients in these lineage all differed by at least 30 SNVs. The exception was, in the TUR sub-lineage, two MDR strains isolated from different patients differed by only 18 SNVs. However, the cross-sectional nature of patient recruitment and the lack of epidemiological and demographic data on the patients hampered our ability to infer chains of inter-patient transmission.

The reasons for the selection and dissemination of the Cameroon genotype in West Africa remain unclear, highlighting the need to investigate its evolutionary origins through WGS [10, 12]. In Ghana, the Cameroon clade is believed to have been introduced through the south of the country, where a higher proportion of infection is associated with this clade than in the north [39]. To our knowledge this is the largest collection of genome sequences from the Cameroon clade that has been analysed. This study suggests that this genotype is on the rise and is an important source of MDR-TB in Southwest Nigeria. Insights into the evolution of MTBC lineages that are endemic to West Africa are rare but more will emerge as more genomic epidemiology studies are performed in the region.

*M*. *africanum* remains an important cause of tuberculosis in West Africa [12, 39] despite reports of a diminishing presence in some regions [40]. Our findings suggest that multidrug resistant MAF1 isolates have been transmitted within this setting during the recent past. The prevalence of the *M*. *africanum* sub-lineage MAF1 in this dataset (9.5%) is lower than a recent estimate [12] and this may be due to variable prevalence of MAF1 in different regions of the country.

We report genomic changes that led to worsening drug resistance phenotypes through acquisition of resistance mutations. These findings underscore the adverse consequences of delayed diagnosis of resistance, ineffective treatment, and treatment interruptions or failure. Nigeria has implemented the WHO recommended Directly Observed Treatment Short Course (DOTS) since 1996. Despite efforts to control TB, interruption of treatment has been attributed to a number of factors including lack of knowledge of duration of treatment, living far from hospital, inability to afford transport and abandoning treatment due to feeling better [41]. Going forward, there is a need to investigate the acquisition of drug resistance mutations in West-African lineages, such as the Ghana and Cameroon genotypes.

Early diagnosis of MDR-TB can be instrumental in ensuring treatment success. The WHO recommends rapid testing for at least rifampicin resistance at the time of diagnosis [42]. The implementation of Xpert^**®**^ MTB/RIF (Cepheid Inc.) for rapid detection of *Mycobacterium tuberculosis* complex (MTBC) and rifampicin resistance has been intensified in Nigeria to good effect [43, 44]. However, Xpert^**®**^ needs to be complemented with additional resistance testing [45]. The Hain MTBDR plus kit is a rapid tool for early detection of drug resistance in MTBC using molecular probes. For predicting isoniazid resistance, it has probes that detect the *katG* S315T resistance mutation, as well as resistance mutations in the *inhA* promoter region.

Routine *in situ* use of bacterial whole-genome sequencing is not yet feasible in a low-resource setting like Nigeria for a number of reasons: inconsistent electricity supply, lack of funds for sequencers and reagents and paucity of trained technicians who can perform library preparations, sequencing and downstream bioinformatics analysis. Culture facilities are also scarce, so the most practical method for diagnosis of MDR-TB probably remains genotypic testing by commercially available tests such as the Xpert MTB/RIF and Line Probe Assays. However WGS can contribute towards understanding the epidemiology of MTBC in Nigeria in a research capacity. South Africa, an African country with a comparably sized economy, has managed to make extensive use of WGS of local TB isolates [46].

## Conclusion

Transmission of MDR-TB is an ongoing phenomenon in our study sites in Southwest Nigeria and this requires urgent intervention. Our data suggests that the Cameroon clade is involved in transmission of MDR-TB in this setting. Our study underscores the urgent need for routine drug susceptibility testing in Nigeria and more concerted efforts to ensure that patients are put through the full course of treatment. These measures will give patients a better chance of survival and will help to curb the spread of MDR-TB.

### Study limitations

In the absence of epidemiological and patient demographic information, it was not possible to infer with certainty inter-patient transmission based solely on genome sequences. The small sample size presents a major limitation to our study and our ability to infer conclusions. Our dataset was not powered to infer the population structure or the prevalence of drug resistance in the community since patients were recruited only from referral hospitals—to determine the prevalence of MDR-TB, a *de novo* cross-sectional sampling strategy would have to be implemented. Furthermore the proportion of MDR-TB isolates that have been genome-sequenced is higher than their prevalence because the initial emphasis of this study was on MDR-TB. Nonetheless, we provide evidence of transmission of MDR-TB and worsening drug resistance during the course of treatment from a low-resource, high-TB burden setting. The small sample size limited our ability to detect novel resistance mutations. The resistance prediction was restricted to mutations that were present in the databases used by the three software, if other loci were probed the sensitivity of our methods relative to phenotypic testing might have been improved.

## Supporting information

S1 Table Isolates metadata. Metadata for study isolates including study site, age, sex, treatment status (study case), HIV status, MDR & pre-XDR status, as well as lineage information inferred from kvarq, phyressse (Phy) and TBProfiler (tbp).
(XLSX)

S2 Table Isolate resistance profiles. Table showing phenotypic resistance profiles to first line drugs (rifampicin, isoniazid, streptomycin and ethambutol) for all isolates and to second line drugs (capreomycin, fluoroquinolone, ethionamide and amikacin) for MDR isolates. Table also lists presence of known resistance mutations as detected by the three resistance prediction tools kvarq, phyrese and tbprofiler.
(XLSX)

## Abbreviations

TB: Tuberculosis
MDR-TB: Multidrug resistant tuberculosis
Pre-XDR-TB: Pre-extensively drug resistant tuberculosis
XDR-TB: Extensively drug resistant tuberculosis
MTBC: Mycobacterium tuberculosis complex
WGS: Whole genome sequencing
WANETAM: West African Nodes of Excellence for Tuberculosis AIDS and Malaria
MIRU VNTR: Mycobacterial Interspersed Repetitive Units Variable Number Tandem Repeats
CTAB: Cetyltrimethyl ammonium bromide
MRCG: MRC Unit the Gambia
SNV: Single nucleotide variant
MAF1: Mycobacterium africanum West Africa 1
DOTS: Directly observed treatment short course

## References

[1] Brennan PJ, Nikaido H. The envelope of mycobacteria. Annual review of biochemistry. 1995;64:29–63. doi: 10.1146/annurev.bi.64.070195.000333 . 7574484 10.1146/annurev.bi.64.070195.000333

[2] Gagneux S. Genetic diversity in Mycobacterium tuberculosis. Current topics in microbiology and immunology. 2013;374:1–25. doi: 10.1007/82_2013_329 . 23677208 10.1007/82_2013_329

[3] Comas I, Coscolla M, Luo T, Borrell S, Holt KE, Kato-Maeda M, et al Out-of-Africa migration and Neolithic coexpansion of Mycobacterium tuberculosis with modern humans. Nature genetics. 2013;45(10):1176–82. doi: 10.1038/ng.2744 ; PubMed Central PMCID: PMC3800747. 23995134 10.1038/ng.2744 PMC3800747

[4] Bos KI, Harkins KM, Herbig A, Coscolla M, Weber N, Comas I, et al Pre-Columbian mycobacterial genomes reveal seals as a source of New World human tuberculosis. Nature. 2014;514(7523):494–7. doi: 10.1038/nature13591 ; PubMed Central PMCID: PMC4550673. 25141181 10.1038/nature13591 PMC4550673

[5] Kay GL, Sergeant MJ, Zhou Z, Chan JZ, Millard A, Quick J, et al Eighteenth-century genomes show that mixed infections were common at time of peak tuberculosis in Europe. Nature communications. 2015;6:6717 doi: 10.1038/ncomms7717 ; PubMed Central PMCID: PMC4396363. 25848958 10.1038/ncomms7717 PMC4396363

[6] Galagan JE. Genomic insights into tuberculosis. Nature reviews Genetics. 2014;15(5):307–20. doi: 10.1038/nrg3664 . 24662221 10.1038/nrg3664

[7] Gagneux S. Host-pathogen coevolution in human tuberculosis. Philosophical transactions of the Royal Society of London Series B, Biological sciences. 2012;367(1590):850–9. doi: 10.1098/rstb.2011.0316 ; PubMed Central PMCID: PMC3267123. 22312052 10.1098/rstb.2011.0316 PMC3267123

[8] Coscolla M, Gagneux S. Consequences of genomic diversity in Mycobacterium tuberculosis. Seminars in immunology. 2014;26(6):431–44. doi: 10.1016/j.smim.2014.09.012 ; PubMed Central PMCID: PMC4314449. 25453224 10.1016/j.smim.2014.09.012 PMC4314449

[9] Niobe-Eyangoh SN, Kuaban C, Sorlin P, Cunin P, Thonnon J, Sola C, et al Genetic biodiversity of Mycobacterium tuberculosis complex strains from patients with pulmonary tuberculosis in Cameroon. Journal of clinical microbiology. 2003;41(6):2547–53. doi: 10.1128/JCM.41.6.2547-2553.2003 ; PubMed Central PMCID: PMC156567. 12791879 10.1128/JCM.41.6.2547-2553.2003 PMC156567

[10] Thumamo BP, Asuquo AE, Abia-Bassey LN, Lawson L, Hill V, Zozio T, et al Molecular epidemiology and genetic diversity of Mycobacterium tuberculosis complex in the Cross River State, Nigeria. Infection, genetics and evolution: journal of molecular epidemiology and evolutionary genetics in infectious diseases. 2012;12(4):671–7. doi: 10.1016/j.meegid.2011.08.011 ; PubMed Central PMCID: PMC3369698. 21878397 10.1016/j.meegid.2011.08.011 PMC3369698

[11] Godreuil S, Torrea G, Terru D, Chevenet F, Diagbouga S, Supply P, et al First molecular epidemiology study of Mycobacterium tuberculosis in Burkina Faso. Journal of clinical microbiology. 2007;45(3):921–7. doi: 10.1128/JCM.01918-06 ; PubMed Central PMCID: PMC1829100. 17251410 10.1128/JCM.01918-06 PMC1829100

[12] Lawson L, Zhang J, Gomgnimbou MK, Abdurrahman ST, Le Moullec S, Mohamed F, et al A molecular epidemiological and genetic diversity study of tuberculosis in Ibadan, Nnewi and Abuja, Nigeria. PloS one. 2012;7(6):e38409 doi: 10.1371/journal.pone.0038409 ; PubMed Central PMCID: PMC3377642. 22723859 10.1371/journal.pone.0038409 PMC3377642

[13] Yeboah-Manu D, Asante-Poku A, Bodmer T, Stucki D, Koram K, Bonsu F, et al Genotypic diversity and drug susceptibility patterns among M. tuberculosis complex isolates from South-Western Ghana. PloS one. 2011;6(7):e21906 doi: 10.1371/journal.pone.0021906 ; PubMed Central PMCID: PMC3133566. 21779354 10.1371/journal.pone.0021906 PMC3133566

[14] de Jong BC, Antonio M, Gagneux S. Mycobacterium africanum—review of an important cause of human tuberculosis in West Africa. PLoS neglected tropical diseases. 2010;4(9):e744 doi: 10.1371/journal.pntd.0000744 ; PubMed Central PMCID: PMC2946903. 20927191 10.1371/journal.pntd.0000744 PMC2946903

[15] Eurosurveillance editorial t. WHO publishes Global tuberculosis report 2013. Euro surveillance: bulletin Europeen sur les maladies transmissibles = European communicable disease bulletin. 2013;18(43). . 24176622

[16] WHO. Global Tuberculosis Report 2016. WHO Library Cataloguing-in-Publication Data: 2016.

[17] Musa BM, Musa B, Muhammed H, Ibrahim N, Musa AG. Incidence of tuberculosis and immunological profile of TB/HIV co-infected patients in Nigeria. Annals of thoracic medicine. 2015;10(3):185–92. doi: 10.4103/1817-1737.160838 ; PubMed Central PMCID: PMC4518349. 26229561 10.4103/1817-1737.160838 PMC4518349

[18] WHO. Global tuberculosis report 2015. 2015.

[19] Lawson L, Habib AG, Okobi MI, Idiong D, Olajide I, Emenyonu N, et al Pilot study on multidrug resistant tuberculosis in Nigeria. Annals of African medicine. 2010;9(3):184–7. doi: 10.4103/1596-3519.68355 . 20710112 10.4103/1596-3519.68355

[20] Falzon D, Mirzayev F, Wares F, Baena IG, Zignol M, Linh N, et al Multidrug-resistant tuberculosis around the world: what progress has been made? The European respiratory journal. 2015;45(1):150–60. doi: 10.1183/09031936.00101814 ; PubMed Central PMCID: PMC4318660. 25261327 10.1183/09031936.00101814 PMC4318660

[21] Koser CU, Bryant JM, Becq J, Torok ME, Ellington MJ, Marti-Renom MA, et al Whole-genome sequencing for rapid susceptibility testing of M. tuberculosis. The New England journal of medicine. 2013;369(3):290–2. doi: 10.1056/NEJMc1215305 ; PubMed Central PMCID: PMC3836233. 23863072 10.1056/NEJMc1215305 PMC3836233

[22] Walker TM, Kohl TA, Omar SV, Hedge J, Del Ojo Elias C, Bradley P, et al Whole-genome sequencing for prediction of Mycobacterium tuberculosis drug susceptibility and resistance: a retrospective cohort study. The Lancet Infectious diseases. 2015 doi: 10.1016/S1473-3099(15)00062-6 . 26116186 10.1016/S1473-3099(15)00062-6 PMC4579482

[23] Kendall EA, Fofana MO, Dowdy DW. Burden of transmitted multidrug resistance in epidemics of tuberculosis: a transmission modelling analysis. The Lancet Respiratory medicine. 2015;3(12):963–72. doi: 10.1016/S2213-2600(15)00458-0 ; PubMed Central PMCID: PMC4684734. 26597127 10.1016/S2213-2600(15)00458-0 PMC4684734

[24] Hatherell HA, Colijn C, Stagg HR, Jackson C, Winter JR, Abubakar I. Interpreting whole genome sequencing for investigating tuberculosis transmission: a systematic review. BMC medicine. 2016;14:21 doi: 10.1186/s12916-016-0566-x ; PubMed Central PMCID: PMC4804562. 27005433 10.1186/s12916-016-0566-x PMC4804562

[25] Walker TM, Ip CL, Harrell RH, Evans JT, Kapatai G, Dedicoat MJ, et al Whole-genome sequencing to delineate Mycobacterium tuberculosis outbreaks: a retrospective observational study. The Lancet Infectious diseases. 2013;13(2):137–46. doi: 10.1016/S1473-3099(12)70277-3 ; PubMed Central PMCID: PMC3556524. 23158499 10.1016/S1473-3099(12)70277-3 PMC3556524

[26] Kato-Maeda M, Ho C, Passarelli B, Banaei N, Grinsdale J, Flores L, et al Use of whole genome sequencing to determine the microevolution of Mycobacterium tuberculosis during an outbreak. PloS one. 2013;8(3):e58235 doi: 10.1371/journal.pone.0058235 ; PubMed Central PMCID: PMC3589338. 23472164 10.1371/journal.pone.0058235 PMC3589338

[27] Lee RS, Radomski N, Proulx JF, Manry J, McIntosh F, Desjardins F, et al Reemergence and amplification of tuberculosis in the Canadian arctic. The Journal of infectious diseases. 2015;211(12):1905–14. doi: 10.1093/infdis/jiv011 . 25576599 10.1093/infdis/jiv011

[28] Roetzer A, Diel R, Kohl TA, Ruckert C, Nubel U, Blom J, et al Whole genome sequencing versus traditional genotyping for investigation of a Mycobacterium tuberculosis outbreak: a longitudinal molecular epidemiological study. PLoS medicine. 2013;10(2):e1001387 doi: 10.1371/journal.pmed.1001387 ; PubMed Central PMCID: PMC3570532. 23424287 10.1371/journal.pmed.1001387 PMC3570532

[29] Guerra-Assuncao JA, Crampin AC, Houben RM, Mzembe T, Mallard K, Coll F, et al Large-scale whole genome sequencing of M. tuberculosis provides insights into transmission in a high prevalence area. eLife. 2015;4 doi: 10.7554/eLife.05166 ; PubMed Central PMCID: PMC4384740. 25732036 10.7554/eLife.05166 PMC4384740

[30] Gehre F, Otu J, Kendall L, Forson A, Kwara A, Kudzawu S, et al The emerging threat of pre-extensively drug-resistant tuberculosis in West Africa: preparing for large-scale tuberculosis research and drug resistance surveillance. BMC medicine. 2016;14(1). doi: 10.1186/s12916-016-0704-5 27806714 10.1186/s12916-016-0704-5 PMC5094099

[31] Ardito F, Posteraro B, Sanguinetti M, Zanetti S, Fadda G. Evaluation of BACTEC Mycobacteria Growth Indicator Tube (MGIT 960) automated system for drug susceptibility testing of Mycobacterium tuberculosis. Journal of clinical microbiology. 2001;39(12):4440–4. doi: 10.1128/JCM.39.12.4440-4444.2001 ; PubMed Central PMCID: PMC88562. 11724858 10.1128/JCM.39.12.4440-4444.2001 PMC88562

[32] Barrera L, Cooreman E, de Dieu Iragena J, Drobniewski F, Duda P, Havelkova M, et al. Policy Guidance on Drug-Susceptibility Testing (DST) of Second-Line Antituberculosis Drugs. WHO Guidelines Approved by the Guidelines Review Committee. Geneva2008. 26290924

[33] Somerville W, Thibert L, Schwartzman K, Behr MA. Extraction of Mycobacterium tuberculosis DNA: a question of containment. Journal of clinical microbiology. 2005;43(6):2996–7. doi: 10.1128/JCM.43.6.2996-2997.2005 ; PubMed Central PMCID: PMC1151963. 15956443 10.1128/JCM.43.6.2996-2997.2005 PMC1151963

[34] Witney AA, Gould KA, Arnold A, Coleman D, Delgado R, Dhillon J, et al Clinical application of whole-genome sequencing to inform treatment for multidrug-resistant tuberculosis cases. Journal of clinical microbiology. 2015;53(5):1473–83. doi: 10.1128/JCM.02993-14 ; PubMed Central PMCID: PMC4400773. 25673793 10.1128/JCM.02993-14 PMC4400773

[35] Stamatakis A. RAxML version 8: a tool for phylogenetic analysis and post-analysis of large phylogenies. Bioinformatics. 2014;30(9):1312–3. doi: 10.1093/bioinformatics/btu033 ; PubMed Central PMCID: PMC3998144. 24451623 10.1093/bioinformatics/btu033 PMC3998144

[36] Coll F, McNerney R, Preston MD, Guerra-Assuncao JA, Warry A, Hill-Cawthorne G, et al Rapid determination of anti-tuberculosis drug resistance from whole-genome sequences. Genome medicine. 2015;7(1):51 doi: 10.1186/s13073-015-0164-0 ; PubMed Central PMCID: PMC4446134. 26019726 10.1186/s13073-015-0164-0 PMC4446134

[37] Feuerriegel S, Schleusener V, Beckert P, Kohl TA, Miotto P, Cirillo DM, et al PhyResSE: a Web Tool Delineating Mycobacterium tuberculosis Antibiotic Resistance and Lineage from Whole-Genome Sequencing Data. Journal of clinical microbiology. 2015;53(6):1908–14. doi: 10.1128/JCM.00025-15 ; PubMed Central PMCID: PMC4432036. 25854485 10.1128/JCM.00025-15 PMC4432036

[38] Steiner A, Stucki D, Coscolla M, Borrell S, Gagneux S. KvarQ: targeted and direct variant calling from fastq reads of bacterial genomes. BMC genomics. 2014;15:881 doi: 10.1186/1471-2164-15-881 ; PubMed Central PMCID: PMC4197298. 25297886 10.1186/1471-2164-15-881 PMC4197298

[39] Yeboah-Manu D, Asare P, Asante-Poku A, Otchere ID, Osei-Wusu S, Danso E, et al Spatio-Temporal Distribution of Mycobacterium tuberculosis Complex Strains in Ghana. PloS one. 2016;11(8):e0161892 doi: 10.1371/journal.pone.0161892 ; PubMed Central PMCID: PMC5001706. 27564240 10.1371/journal.pone.0161892 PMC5001706

[40] Koro Koro F, Kamdem Simo Y, Piam FF, Noeske J, Gutierrez C, Kuaban C, et al Population dynamics of tuberculous Bacilli in Cameroon as assessed by spoligotyping. Journal of clinical microbiology. 2013;51(1):299–302. doi: 10.1128/JCM.01196-12 ; PubMed Central PMCID: PMC3536219. 23115266 10.1128/JCM.01196-12 PMC3536219

[41] Ibrahim LM, Hadejia IS, Nguku P, Dankoli R, Waziri NE, Akhimien MO, et al Factors associated with interruption of treatment among Pulmonary Tuberculosis patients in Plateau State, Nigeria. 2011. The Pan African medical journal. 2014;17:78 doi: 10.11604/pamj.2014.17.78.3464 ; PubMed Central PMCID: PMC3972906. 24711884 10.11604/pamj.2014.17.78.3464 PMC3972906

[42] WHO. WHO treatment guidelines for drug-resistant tuberculosis—2016. 2016.

[43] Abdurrahman ST, Emenyonu N, Obasanya OJ, Lawson L, Dacombe R, Muhammad M, et al The hidden costs of installing Xpert machines in a tuberculosis high-burden country: experiences from Nigeria. The Pan African medical journal. 2014;18:277 doi: 10.11604/pamj.2014.18.277.3906 ; PubMed Central PMCID: PMC4258200. 25489371 10.11604/pamj.2014.18.277.3906 PMC4258200

[44] Ani A, Isah Y, Pwol R, Lekuk C, Ashi-Sulaiman T, Akindgh M, et al Detection of Mycobacterium tuberculosis by rapid molecular methods augments acid fast bacilli (AFB) smear microscopy in a non-culture tuberculosis laboratory. African Journal of Microbiology Research. 2015;9(30):960–4. Epub 01 April 2015. doi: 10.5897/AJMR2014.7358

[45] Drobniewski F, Nikolayevskyy V, Maxeiner H, Balabanova Y, Casali N, Kontsevaya I, et al Rapid diagnostics of tuberculosis and drug resistance in the industrialized world: clinical and public health benefits and barriers to implementation. BMC medicine. 2013;11:190 doi: 10.1186/1741-7015-11-190 ; PubMed Central PMCID: PMC3765611. 23987891 10.1186/1741-7015-11-190 PMC3765611

[46] Cohen KA, Abeel T, Manson McGuire A, Desjardins CA, Munsamy V, Shea TP, et al Evolution of Extensively Drug-Resistant Tuberculosis over Four Decades: Whole Genome Sequencing and Dating Analysis of Mycobacterium tuberculosis Isolates from KwaZulu-Natal. PLoS medicine. 2015;12(9):e1001880 doi: 10.1371/journal.pmed.1001880 ; PubMed Central PMCID: PMC4587932. 26418737 10.1371/journal.pmed.1001880 PMC4587932

